# A Rare Case Report of 17q23.1q23.2 Microdeletion With Homozygosity of 11p11.2q13.4 in a Newborn

**DOI:** 10.7759/cureus.23290

**Published:** 2022-03-18

**Authors:** Sindhu Barola, Allison M Parrill, Samaan Mahmoudzadeh, Peyman Bizargity, Rita Verma

**Affiliations:** 1 Pediatrics, Nassau University Medical Center, East Meadow, USA; 2 Clinical Medicine, American University of the Caribbean School of Medicine, Cupecoy, SXM; 3 Basic Sciences, American University of the Caribbean School of Medicine, Cupecoy, SXM; 4 Medical Genetics, Northwell Health, Great Neck, USA; 5 Neonatology, Nassau University Medical Center, East Meadow, USA

**Keywords:** neonatal, intensive care units, pediatrics, genetic diseases inborn, chromosome deletion

## Abstract

We present the case of a newborn with 17q23.1q23.2 microdeletion and additional homozygosity of 11p11.2q13.4. In the literature, 17q23.1q23.2 microdeletion syndrome is a novel syndrome reported in nine patients. Our patient is a full-term baby boy admitted to a neonatal intensive care unit for hypoglycemia, respiratory distress, presumed sepsis, and thrombocytopenia. General appearance revealed microcephaly, micrognathia, ankyloglossia, small mouth, and high arch palate. The patient also presented with hypotonia, poor feeding, and poor weight gain in the first week of life followed by hypertonia and tremors from the second week of life. The phenotypic and clinical presentation lead to the genetic investigation of microarray which revealed 17q23.1q23.2 microdeletion and additional homozygosity of 11p11.2q13.4.

## Introduction

Across the literature, 17q23.1q23.2 microdeletion syndrome is a novel syndrome reported in nine patients [1-3]. This deletion syndrome occurs more frequently in female patients and presents as general developmental delay, cardiac defects, and various musculoskeletal abnormalities [1,2]. Cardiac defects include patent ductus arteriosus (PDA), atrial septal defect (ASD), bicuspid aortic valves. Several examples of musculoskeletal abnormalities in patients with this syndrome include short stature, long and thin fingers, tibial torsion, aberrant acetabulum, and femoral head ossification, scoliosis, and limited extension of knee and elbow joints [1]. Additional abnormalities, such as microcephaly, low birth weight, postnatal growth retardation, and hearing loss presented in several cases [1,2], and multiple cases with pulmonary hypertension [1,3]. One newborn female expired at 14 hours of life from complications due to pulmonary hypertension and lung hypoplasia [3]. Varying clinical presentations of 17q23.1q23.2 microdeletion syndrome suggests the involvement of several genes [1-3].

Literature review suggests that genes residing within the 2.2 Mb 17q23.1q23.2 microdeletion potentially influence limb maturation [1,4-9] and atrial and ventricular development [10]. Additionally, researchers discovered that genes affected by microdeletion predispose patients to sensorineural hearing loss [2]. A recent study proposed these genes also affect lung development leading to lung hypoplasia and pulmonary hypertension [3, 11]. More specifically, members of the T-box (TB) family transcription factors, *TBX2*, *TBX4*, and *TBX6*, represent important regulators of embryonic development in vertebrates [3,12]. However, thus far these genes and their mechanisms are not rigorously studied within human models. 

Here we describe a rare case of 17q23.1q23.2 microdeletion with homozygosity of 11p11.2q13.4. Our objective is to further characterize the phenotypic associations of this rare genetic condition.

## Case presentation

A newborn Hispanic male was admitted to the neonatal intensive care unit (NICU) for respiratory distress, hypoglycemia, and presumed sepsis. He was delivered via Cesarean Section (C-section) for non-reassuring fetal heart rate (NRFHR) consisting of minimal variability, late decelerations, and a Biophysical Profile Test (BPP) of 4/10. Membranes ruptured at delivery which was complicated by the nuchal cord wrapping around the neonate’s body and arm twice, and thick, meconium-stained amniotic fluid. Umbilical cord gas revealed a pH of 6.99. The mother’s clinical findings indicate she is Blood Type A+, Antibody Negative. The mother’s blood was positive for Rubella antibody. All other maternal infectious laboratory results were negative: Hepatitis B/HIV/Treponema pallidum, Neisseria gonorrhoeae/Chlamydia trachomatis (GC/CT), and Group-B Streptococcus (GBS). He was born at 39 weeks gestation, weighed 3090 grams, length of 53.5 cm (~90 percentile), head circumference of 32.5 cm (<10th percentile), and appeared appropriate for gestational age with microcephaly. Apgar scores of six and eight were recorded at one and five minutes, respectively. The patient required positive pressure ventilation (PPV) for one minute and continuous positive airway pressure (CPAP) for 10 minutes intermittently for nasal flaring and pulse oximetry measuring 92-94% saturation. After respiratory improvement, the patient was transferred to the newborn nursery on room air. While in the nursery, he presented with nasal flaring and decreased pulse oximetry (low 80’s percentage), low blood glucose (35 mg/dL and 22 mg/dL), and poor tone. He was transferred to the NICU for respiratory distress and hypoglycemia. He was immediately administered one bolus of Dextrose 10%, and one bolus of Normal Saline (NS) for low mean arterial pressure (MAP). In the NICU, the patient was transitioned from CPAP to bilevel positive airway pressure (BiPAP) for persistent grunting and increased work of breathing on day two of life. He transitioned to room air on day two of life. Chest X-ray was suspicious of Respiratory Distress Syndrome (RDS). He was on antibiotics until blood cultures returned negative for two days. TORCH (Toxoplasma, Rubella, Cytomegalovirus, Herpes) antibodies work-up was done to rule out the infectious causes of microcephaly. His blood glucose improved and he was placed on maintenance intravenous (IV) fluids for two days later transitioned to full feeds on day three. Intravenous immunoglobulin (IVIG) and platelets were administered for suspected autoimmune thrombocytopenia. The patient remained in the NICU for poor weight gain and feeding. 

Right eye infection with *Enterococcus faecalis* further complicated the NICU course. Ciprofloxacin eye drops (0.3%) were administered for one week. An eye exam was performed given the family history of Retinitis Pigmentosa and the eye exam was normal. Brain magnetic resonance imaging (MRI) reported relative under-myelination potentially appropriate for the patient's age and electroencephalogram (EEG) revealed abnormal spikes. Chromosomal microarray revealed a 17q23.1q23.2 microdeletion and an homozygosity of 11p11.2q13.4. Specifically, the microarray analysis revealed a 1.8 Mb interstitial deletion of genes *DHX40*, *CLTC*, *PTRH2*, *VMP1*, *MIR21*, *TUBD1*, *RPS6KB1*, *RNFT1*, *TBC1D3P1-DHX40P1*, *LOC101927755*, *MIR4737*, *HEATR6*, *LOC105371849*, *WFDC21P*, *LOC653653*, *CA4*, *USP32*, *SCARNA20*, *C17orf64*, *APPBP2*, *LOC388406*, *PPM1D*, and *BCAS*. Surgery evaluated him for ankyloglossia and stated surgical repair was not recommended. The urology team evaluated him for Glandular hypospadias and recommended follow-up in six months. Radiographic imaging of the right hand and right knee showed no abnormalities in structure, shape, size, mineralization or ossification of hand and patellar bones. To rule out cardiac anomalies, an Echocardiogram was performed which showed no heart defects. Renal sonogram was performed to rule out associated anomalies which were normal. Clinical presentation is described in Table 1. 

**Table 1:** Patient Clinical Features and Physical Examination Findings Electroencephalogram (EEG), Electrocardiogram (EKG)

System	Clinical Features
General Appearance	Microcephaly, micrognathia, ankyloglossia, small mouth, high-arched palate, mild hypertelorism, smooth philtrum, thin upper lip.
Respiratory	Respiratory Distress Syndrome at birth
Cardiac	Early systolic murmur grade I-II/VI along the left sternal border.No rubs or gallops; Normal Echo at birth; Mild left branch pulmonary stenosis at 6 weeks; Normal EKG
Genitourinary	Normal abdominal ultrasound; Glandular hypospadias
Musculoskeletal	No ossification or mineralization defects on patellar and hand X-ray; Long, thin fingers. Negative Ortolani and Barlow tests.
Hearing	No hearing loss on newborn screening Auditory Brainstem Response test.
Eyesight	Normal red reflex
Hematological	Thrombocytopenia and hyperbilirubinemia
Neurological	Hypotonia, hypertonia, and tremors; Abnormal spikes on EEG Startle at 6 weeks follow-up

At the six week neurology follow-up, the patient presented with hypertonia, a decreased Moro reflex, and normal electroencephalogram (EEG). During the six week cardiology follow-up, the patient presented with an early systolic, grade I-II/VI murmur along the left sternal border, a normal electrocardiogram (EKG), and a mild left branch pulmonary stenosis. At eight week follow-up, the patient presented with diminished reflexes. At 10 week follow-up, the patient weight and head circumference recorded <2nd percentile despite parental reported adequate formula intake. Patient was referred to Pediatric Gastroenterology for failure to thrive. At three month follow-up, the patient was gaining weight and referred to Otolaryngology for a palatal ulcer. Early intervention was initiated with the patient including physiotherapy. At six month follow-up, the patient was gaining weight appropriately, and following a growth curve. 

## Discussion

The current report presented the tenth case, and the second male with 17q23.1q23.2 microdeletion syndrome. Current literature describes a small number of cases. The estimated prevalence is 0.015% based on the presence of 17q23.1q23.2 microdeletion in three of 19,912 patients' genomic microarrays between November 2007 and October 2009 [1,13]. Within our patient, the microarray reported multiple autosomal dominant (AD) Online Mendelian Inheritance in Man (OMIM), Protein Phosphate Magnesium Manganese-Dependent 1D (*PPM1D*) (*605100), and Clathrin Heavy Chain (*CLTC*) (*118955) gene deletions. These deletions will likely result in developmental and phenotypic abnormalities. The autosomal dominant neurodevelopmental disorder, Jansen-de Vries syndrome (OMIM #617450), involves *PPM1D* de novo loss-of-function variants. This syndrome presents as behavioral abnormalities, delayed psychomotor development, and intellectual disability with speech delay. Many patients exhibit additional features including gastrointestinal difficulties, high pain tolerance, sound hypersensitivity, facial abnormalities, strabismus, and small hands and feet [14,15]. AD intellectual disability 56 (OMIM #617854) associates with de novo loss-of-function variants of *CLTC*. Our patient’s deletion interval partially overlaps the 17q23.1q23.2 recurrent region of chromosome 17q23.1-q23.2 deletion syndrome (OMIM #613355). However, the critical genes *TBX2* and *TBX4* remain outside the deleted segment. Individuals with autosomal dominant retinitis pigmentosa 17 (OMIM #600852) presented with heterozygous sequence variants of *CA4* (*114760). Additionally, the patient’s father and uncle possessed a history of retinitis pigmentosa diagnosed at ages 19 and 14 years, respectively. Patients suffering from infantile-onset multisystem neurologic, endocrine, and pancreatic disease (#616263) carried biallelic sequence variants of *PTRH2 *(*608625). These mutations may account for the neurologic abnormalities, such as hypertonia and decreased Moro reflex, presenting in the patient. Furthermore, the microarray analysis revealed an approximately 29 Mb region of allelic homozygosity on chromosome 11 within our patient. However, the clinical significance of homozygosity in this region currently remains unclear. Maternal uniparental disomy (UPD) 11 is associated with rare cases of Silver-Russell syndrome (OMIM #180860). This disease presents with severe intrauterine growth restriction, poor postnatal growth, dysmorphic facial features, and body asymmetry. On the other hand, Paternal UPD 11 is associated with the Beckwith-Wiedemann syndrome (BWS; #130650) or a more severe phenotype than maternal UPD. As an overgrowth disorder, BWS predisposes afflicted individuals to tumor development. Our patient’s microarray report indicates the loss of many genes on chromosome 17, and a region of homozygosity on chromosome 11 which may impact future growth and development. Lastly, Homozygosity of 11p11.2q13.4 has been associated with delayed psychomotor development, absent speech, severe intellectual disability, and postnatal microcephaly, with brain malformations [16].

In comparison to the previously published nine cases on 17q23.1q23.2 microdeletion, our patient showed no evidence of ASD, PDA, pulmonary artery hypertension, ossification or mineralization defects, or hearing loss. However, despite early normal Echos, the patient was diagnosed with left branch pulmonary stenosis at six weeks of age. The current case presented with long, thin fingers and microcephaly similar to findings in previously reported cases. New findings within the current case include microcephaly, micrognathia, ankyloglossia, small mouth, high arch palate, glandular hypospadias, and thrombocytopenia. He presented with hypotonia, poor feeding, poor weight gain in the first week of life followed by hypertonia, and tremors from the second week of life. Neurology managed the hypertonia and tremors, and Urology the glandular hypospadias. The other male case presented with a shawled scrotum [1]. In comparison to a previously published case on 11p11.2q13.4 [16], our patient presented with hypertonia, microcephaly, high arched palate, and startle reflex at two months. In conclusion, the current case presented both new and typical features of the 17q23.1q23.2 microdeletion syndrome and identified homozygosity of 11p11.2q13.4 at an early stage. 

The authors speculate that heterogeneity in the microdeletion breakpoint may partially explain variations across phenotypes of these cases. Furthermore, genetic associations may exist that are not yet discovered within 17q23.1q23.2 microdeletion syndrome, or interval deletions in this genetic region. As such, additional characteristics related to our patient’s microdeletion may occur later in development. For example, a genome linkage analysis discovered loci associated with Multiple Sclerosis (MS) in the 17q23.1q23.2 microdeletion region [15]. Cases with 17q23.1q23.2 microdeletion syndrome potentially alter the risk of developing MS throughout the lifespan as compared to the general population. 

The mode of inheritance of 17q23.1q23.2 microdeletion syndrome remains undetermined. A case series identified de novo 17q23.1q23.2 microdeletion based on parental fluorescent in situ hybridization genotyping results in five of seven individuals [1]. In a single case, the microdeletion presented as de novo mutation [2]. Another case identified the de novo 17q23.1q23.2 microdeletion on maternally inherited chromosomes [3]. Parental microarray was recommended, however, it was not performed in the patient's family. 

Future research may further characterize 17q23.1q23.2 microdeletion syndrome to potentially generate diagnostic testing guidelines for associated anomalies. Additionally, whole-genome sequencing of patients with this microdeletion may lead to a better understanding of potential gene interaction. The authors anticipate that such knowledge may improve detection, treatment, support, and patient outcomes related to this specific microdeletion syndrome. 

## Conclusions

The current case highlights a rare genetic microdeletion in a male newborn. As there are only a small number of cases published to date, disease progression and management of this genetic microdeletion remains unclear. The use of advanced genetic screening may diagnose more cases of 17q23.1q23.2 microdeletion in newborns and generate further characterization of the disease. The addition of detailed patient cases may lead to pertinent screening and treatment guidelines may be produced to help patients, families, and their providers manage 17q23.1q23.2 microdeletion syndrome.
